# Editorial commentary: Native mitral valve infective endocarditis—Surgical concerns?

**DOI:** 10.1111/jocs.16892

**Published:** 2022-08-30

**Authors:** Amila Kahrovic, Philipp Angleitner, Martin Andreas, Paul Werner, Marco Russo

**Affiliations:** ^1^ Department of Cardiac Surgery Medical University of Vienna Vienna Austria; ^2^ Department of Cardiac Surgery and Heart Transplantation San Camillo Forlanini Hospital of Rome Rome Italy

Infective endocarditis (IE) is still associated with high rates of mortality and morbidity despite substantial improvements in medical and surgical management during the last decades.^1^ Surgery is primarily recommended for patients presenting with heart failure, extensive valvular and/or paravalvular lesions, and those at high risk of embolism.^2^ In this setting, the preferred surgical procedure is debated since surgeons are often faced with a large variety of pathological patterns. Surgical procedures predominantly performed comprise mitral valve repair (MVP) and mitral valve replacement (MVR). MVP is a technically demanding procedure necessitating substantial surgical expertise to preserve the integrity and functionality of the mitral valve.^2^, ^3^ The wide armamentarium of currently available MVP techniques makes this surgical procedure feasible even in cases of challenging lesions.^4^ However, MVR is performed in cases of irreparable damages of the valve leaflets as well as extensively destructive paravalvular lesions.^2^

The present article by Lie et al. assessed superiority between MVP and MVR for active IE of the mitral valve. The decision‐making process for optimal surgical procedure as well as the complexities of surgical techniques for MVP were evaluated. This retrospective study included 187 adult patients who underwent MVP (60.4%) or MVR (39.6%). The logistic regression analysis identified the poor valve quality, large defect following debridement, and heavy valve infection as independent risk factors in favor of the replacement technique. Interestingly, after propensity score matching analysis, the type of surgical procedure (MVP vs. MVR) had no impact on major postoperative complications, short‐term mortality, and midterm mortality. However, the reoperation rate after MVP was significantly higher (*p* = .048), as demonstrated by the Kaplan−Meier analysis. In addition, individual analysis of patients undergoing MVP was performed concerning the repair technique used (complex vs. simple MVP subgroup). The reoperation rate was significantly higher among those who underwent complex repair technique (*p* = .020). The authors concluded that the choice of surgical procedure is driven rather by intraoperative inspection of the infected tissue than the clinical presentation of the patient or severity of infection at baseline.

The findings reported by Lie et al. are of great importance in resolving the controversy about the preferred type of surgical procedure and should be kept in mind in the scope of IE. On this point, we would have some remarks.

IE manifests often with variable pathological spectrums, including both the valve itself and perivalvular tissue, for example, abscess, pseudoaneurysm, or intracardiac fistulas.^5^ Perivalvular lesions are most commonly encountered in instances of uncontrolled infection.^2^ As evidenced by the study of David et al.,^6^ the paravalvular abscess was found to be an independent predictor of operative mortality. Rigorous resection of all infected and devitalized tissue is the gold‐standard approach in patients presenting with IE.^7^, ^8^ Otherwise, the remaining contaminated tissue may be associated with a substantial risk of endocardits recurrence.^9^ The decision‐making process to discriminate between surgical procedures (MVP vs. MVR) is ambiguous and undoubtedly involves more than “eyeballing” the infected tissue. Further, the choice of surgical procedure is guided by the comorbidities of the patient, the severity of the disease, and the grade of acuity, all of which are recognized as powerful drivers of operative mortality.^10^ In the present study, the highlights in the decision‐making process in favor of MVR were poor valve quality, large defects after debridement, and heavy valve infection. The impact of these factors on the surgical procedure is unquestionable and should not be underestimated. However, in cases where tissue destruction is advanced, the feasibility and durability of MVR is a major issue.

The MVP was performed in the present study more frequently than the MVR. Although the literature suggests an increase in the rate of MVP over time, MVR is still considered the predominant surgical strategy.^11^, ^12^ In addition, in the context of MVR surgery, the authors did not address the type of implanted prosthesis (mechanical or biological valve prosthesis). Several analyses demonstrated improved outcomes following implantation of a mechanical valve prosthesis in patients presenting with IE.^13^, ^14^, ^15^ The time of surgery is another concern that needs to be taken into mind for the feasibility of MVP, implying that early surgery might prevent further tissue damage.^16^ Additionally, early surgery for active IE is associated with improved outcomes, as demonstrated in the analysis by Kang et al.^17^

With regard to mortality, the authors of the present study reported no difference in short‐term or medium‐term mortality among study groups. A study by Ruttmann et al.^18^ addressed the superiority of surgical procedures for mitral valve endocarditis and found no difference in 5‐year survival between MVR and MVP. In contrast, Toyoda et al.^12^ found that the MVP is associated with improved outcomes, based on a study of 1970 patients with active IE undergoing MVR versus MVP.

Nevertheless, the durability of the MVP has been examined in several analyses demonstrating inconsistent findings. The long‐term durability of the MVP was reported in a study conducted by Solari et al. (freedom from reoperation at 10 and 15 years of 88% and 81%, respectively).^19^ Tepsuwan et al.^20^ found no differences in reoperation rate between MVP and MVR. Surprisingly, the authors of the present study found a significantly higher reoperation rate for MVP than MVR. For the complex MVP procedure, due to the extensive tissue lesions, the pericardial patch reconstruction technique is most commonly used.^19^, ^21^, ^22^ However, the durability of a pericardial patch in this clinical setting is still uncertain.

Summarizing, each patient has a unique clinical presentation as well as pathological characteristics specific to IE. An individual patient‐tailored surgical strategy is crucial in attaining favorable results. MVR is reasonable to allow full removal of infected tissue, and MVP should not be stressed if it cannot be performed with a high likelihood of success.
